# Barriers to Anti-Hypertensive Medication Adherence Among Patients in Private Healthcare in Edenvale, South Africa

**DOI:** 10.3390/healthcare13182267

**Published:** 2025-09-10

**Authors:** Bernard Hope Taderera

**Affiliations:** Faculty of Health Sciences, Department of Environmental Health, University of Johannesburg, Johannesburg 2001, South Africa; btaderera@uj.ac.za; Tel.: +27-11-559-6254

**Keywords:** adherence, anti-hypertensive medication, barriers

## Abstract

**Background:** Hypertension is a major global public health problem whose prevalence is increasing across the world. In consideration of this, there is insufficient understanding of the barriers that hinder the taking of anti-hypertensive medication among patients. In this regard, the aim of this study was to analyze the possible barriers undermining adherence to anti-hypertensive medication among patients in private healthcare in Edenvale, South Africa. **Methodology:** This study used an exploratory cross-sectional research design within which quantitative data were collected through an online survey on a sample of randomly selected hypertensive patients attending private healthcare facilities in Edenvale. Participation in this study was voluntary, and informed consent was sought from each participant. Anonymity was assured during data collection through the de-identification of respondents and any data about them. The collected data were subjected to descriptive statistical analysis. **Results:** One hundred and twenty-two patients participated in this study. From this, 34.4% of participants revealed that lack of awareness was a barrier to a very small extent. Forgetfulness was a possible barrier to adherence to a large extent amongst 16.4% of participants, and 26.2% of the respondents had, to a large extent, doubts about their hypertension diagnosis. However, 42.6% revealed that side effects and difficulty taking medication whilst away from home (47.5%) were a barrier to a small extent. The fear of side effects (19.7%), interference of alcohol or drug use (29.5%) were challenges to a moderate extent. **Conclusions:** The findings of this study support that hypertensive patients in private healthcare encounter financial constraints, occasionally forget to take their medication, doubt their hypertension diagnosis, and lack awareness about the benefits of taking anti-hypertensive medication. This may be compounded by patients finding the anti-hypertensive medication regimen too complicated, feeling overburdened by having to take too many pills every day, the complexity of the medication regimens, perceived incorrect diagnosis, and lack of social support from family and friends. Understanding the extent of the barriers encountered by patients in taking anti-hypertension medication may help address adherence challenges, which may help improve health outcomes and lessen the burden on health systems in pursuing Sustainable Development Goal 3 and universal health coverage.

## 1. Introduction

Hypertension, defined as the consistently high pressure of blood flow within vessels, is the leading cause of cardiovascular events and all-cause mortality worldwide. Hypertension bears a correlation with the incidence of cardiovascular and renal detriment [1]. Hypertension, together with pre-hypertension and other hazardously high Blood Pressure (BP), accounts for about 8.5 million deaths around the world, mainly through ischemic heart disease, stroke, other vascular diseases, and renal disease. Hypertension may be diagnosed at community and primary care facilities, and there are a number of useful medical products available at reasonably low prices to help treat people with hypertension and also to help prevent its development into other related diseases [2]. As of 2010, nearly one-third of adults worldwide had hypertension. The increasing prevalence of hypertension is mainly attributed to the growing number of older adults [1]. For instance, by the year 2030, it is projected that one in six individuals globally will have reached the age of 60 or older. Furthermore, by 2050, the worldwide population of individuals aged 60 and older is expected to double, reaching 2.1 billion. Notably, the demographic cohort of individuals aged 80 or older is foreseen to undergo a threefold increase between 2020 and 2050, reaching a total of 426 million [3]. In addition, the preference for unhealthy food options (diets rich in sodium and poor in potassium), smoking, and the absence of exercise are also factors that contribute to the increasing occurrence of hypertension in people [1].

It is important to note that the prevalence of hypertension varies across regions and country income groups, with the World Health Organization (WHO) Africa Region reporting the highest prevalence of hypertension (27%) compared to the lowest (18%) in the WHO Americas Region. It is also worth noting that the number of adults with hypertension increased from 594 million in 1975 to 1.13 billion in 2015, with the increase seen largely in Low- and Middle-Income Countries (LMICs) [4]. It is estimated that the number of hypertensive individuals living in Sub-Saharan Africa (SSA) by 2025 will be 125.5 million (95% CI 111.2 to 162.8 million), which corresponds to an increase of about 68% from 2008 to 2025 [5]. This means that hypertension continues to pose a significant burden on the health systems in SSA. In addition, whilst clinical research has been carried out to help understand the diagnosis, treatment, and control of hypertension around the world, more research focused on SSA is still required. Research on SSA may be enhanced by overcoming stumbling blocks across the care continuum, at individual, provider, and system levels in the healthcare system. In this, it is important for research to focus on the important issue of the contexts within African health systems in order to be able to explore the mechanisms, phenotypes, and treatment responses in native SSA populations [6]. This may also help make it possible to help increase awareness and treatment levels amongst Africans to levels above the current reported 34%, and the control of hypertension to above 6.5% [7]. In South Africa (SA), the number of people who are aware of their hypertension condition, are on treatment, and are in control of hypertension remains lower than the actual, despite the multiple calls to action and strategic roadmaps to close this gap from regional and international bodies. As a result, a recent study reports that fewer than 10% of hypertensive men and only 13% of women in SA are aware of their condition. Among those diagnosed and treated, just 14–21% achieve adequate blood pressure control [8]. In this regard, information on hypertension prevalence, awareness, treatment, and control in multiple countries and different types of communities is necessary to provide a baseline for monitoring and also to inform the development of new strategies for improving hypertension control [9].

To intervene against this, countries holding membership in the African Union (AU) at a meeting in Addis Ababa in 2004 concluded that hypertension is one of the leading health problems in African health systems, after HIV/AIDS. As a result, these AU members resolved that there is a need to formulate, disseminate, and implement interventions that include best practices for hypertension detection, treatment, and control that are affordable, available, and accessible to all, and community-oriented. The World Health Organization (WHO)’s Global Action Plan for the Prevention and Control of Non-Communicable Diseases (NCDs) 2013–2020, extended to 2030 by the World Health Assembly in 2019, initially focusing on reducing premature mortality from four key NCDs (cardiovascular diseases, cancer, chronic respiratory diseases, and diabetes) and their shared risk factors (tobacco use, unhealthy diet, physical inactivity, and harmful use of alcohol), now aims to achieve the Sustainable Development Goal (SDG) target 3.4. This target calls for a one-third reduction in premature deaths from NCDs by 2030. This Global Action Plan for the Prevention and Control of NCDs prescribes that United Nations (UN) member countries need to implement immediate interventions towards preventing and controlling NCDs so as to at least achieve prevalence reduction in high blood pressure by 25%, doing so in consideration of local contexts. In this, health ministries and governments in this UN Political Declaration pledged to commit towards preventing and controlling NCDs by setting up and capacitating multi-sectoral national plans, policies, programs, and projects for interventions. To this end, the Pan-African Society of Cardiology (PASCAR) convened a number of times with the goal of exploring interventions against hypertension in African health systems. In this endeavor, however, PASCAR reported a number of barriers undermining the detection, treatment, and control of hypertension at healthcare system governance, health-service provider, and patient levels [10].

At a patient level, however, there is increasing realization that hypertension remains a challenge primarily due to failure to adhere to anti-hypertensive medication amongst patients. Failure to adhere to treatment medication can occur in three fundamental ways: (i) failure to initiate new prescriptions, (ii) failure to take medication in the way that was prescribed, and (iii) failure to persist with the treatment regimen. This was highlighted by a longitudinal study which found that almost 50% of the 4783 patients included in this study had stopped adhering to their prescribed anti-hypertensive medication within 12 months of starting treatment [11]. Non-adherence to anti-hypertension medication results from barriers which include lack of awareness about the need for uptake, financial constraints, forgetfulness, conflict with cultural beliefs, preference for traditional treatment methods, denial, potential side-effects, failure to understand the language in the medication, the use of alcohol and/or drugs which interferes with treatment, challenges taking medication when away from home, long waiting queues at health facility, and discontinuation of medication after one starts feeling better [12,13,14,15,16,17].

It should be noted that non-adherence to medication has both personal and economic consequences, including negative effects on the patient’s health as well as an increased burden on healthcare services. Furthermore, failure to comply with the treatment plan can lead to poor hypertension control [11,18,19]. Poor adherence to anti-hypertensive medication can have severe consequences, noting that uncontrolled hypertension is associated with poorly controlled BP, which can create cardiovascular problems. Some cases of hypertension can be avoided by changing one’s lifestyle, which may assist in normalizing BP. However, more severe cases of hypertension may require a combination of lifestyle changes and pharmacological treatment [20]. Despite these study findings reported in the literature, there remains a gap in knowledge from research exploring the barriers undermining adherence to hypertension medication amongst patients in private medical care, with a particular focus on Gauteng, South Africa. In this regard, the aim of this study was to analyze barriers to adherence to anti-hypertensive medication in terms of their extent and ranking as reported by adult hypertensive patients accessing private healthcare facilities in the Edenvale area in Gauteng, South Africa. The hypothesis is that understanding the extent of the barriers to anti-hypertensive medication may help inform patient-oriented interventions towards adherence for better health outcomes.

## 2. Materials and Methods

### 2.1. Research Design

An exploratory cross-sectional study design was used in this research. In this, quantitative methods were used to analyze barriers undermining adherence to antihypertensive medication amongst patients attending private healthcare facilities in Edenvale, South Africa [21].

### 2.2. Research Setting

This study was carried out at five private healthcare facilities located in the Edenvale area of the Ekurhuleni Municipality of Gauteng Province, South Africa. Research was conducted over a period of three months, from September to November 2023, to allow enrolment of participants and the data collection process.

### 2.3. Population

The targeted population in this study was adult hypertensive patients attending private healthcare centers in Edenvale. The inclusion criteria for the study population included being 18 years of age and older, formally diagnosed with hypertension, having access to a private healthcare provider in the Edenvale area of Gauteng, South Africa, and being identified through assistance from these health facilities as showing challenges adhering to anti-hypertension medication. Patients visiting private health centers either had private medical insurance or were paying for their visits. The exclusion criteria for this research population included those below 18 years, with a previous diagnosis but have since healed and are no longer on medication, and not diagnosed with hypertension, with no access to private healthcare providers in Edenvale, considering that this study focused on patients in private healthcare as a grey area in the literature. However, if the person was a hypertensive patient of the private health facilities in Edenvale but not showing any challenges with adherence to medication, then they were also excluded from this study [21,22]. Hypertension diagnosis in this study is defined as a medical examination that reveals high blood pressure in individuals attending a health facility. Adherence was defined as the extent to which a patient consistently takes their medications as prescribed by their healthcare provider without any challenges.

### 2.4. Sample Size Determination Procedure

This study recruited 122 participants to increase the statistical power during the analysis. This minimum sample size was verified using an online sample size calculator by Raosoft (Seattle, WA, USA) from the United States of America (2023). The following formula was used for sample size calculation:

n = (Z2*p*(1 − p))/(d2)

where;

- n is the required sample size
- Z is the standard score (1.96 for a 95% confidence level)
- p is the estimated proportion or general global prevalence of hypertension in adult patients (0.3)
- d is the desired margin of error or acceptable level of precision (5% in this case).

### 2.5. Sampling and Data Collection

The researchers enlisted the assistance of independent private healthcare practitioners to identify hypertensive patients with adherence or compliance challenges who then became prospective participants. The prospective participants visiting the private clinics were then given a form to complete at their first visit. This study was explained to them on the form, where they had to agree or disagree to participate in this study. For those who agreed to take part in this study, a number was assigned to a list against their name. The numbers were then randomly selected, using a simple random sampling technique, after which those prospective participants with the corresponding name to the selected number were sent a coded link to the study’s Google Forms. This link contained the Information Consent Form and Questionnaire. In order to access the Questionnaire, the participants had to first complete and sign off the Information Consent Form as a way of providing informed consent. All participants completed the Information Consent Form before completing the Questionnaire. The questionnaire consisted of Likert-rated questions on the possible barriers. In this, the rating categories included the following: to a large extent (all the time); to a moderate extent (sometimes); to a small extent (on a few times); to a very small extent (rarely); and to a very large extent (most of the time). In addition, there were also Likert-rated questions aimed at ranking the possible barriers in terms of the following ranking: first most important, second most important, and third most important. The barriers have to be labelled as possible so as not to discourage disclosure [22].

### 2.6. Data Analysis

The collected data were subjected to descriptive statistical analysis using proportions (percentages) to understand the extent (and ranking) of the barriers, in IBM Statistical Package for Social Sciences (SPSS) for Windows Version 26.0. Armonk, NY, USA: IBM Corp. In this, the proportion was determined by the number against the total, expressed as a percentage, of those who gave a particular response [21,22].

### 2.7. Institutional and Research Ethics Clearance

Permission to carry out this study was sought and obtained from private healthcare facilities in Edenvale, Gauteng, South Africa. Ethics clearance was sought and granted by the Research Ethics Committee (REC Clearance Letter Number REC-1942-2023) of the Faculty of Health Sciences, University of Johannesburg, South Africa.

## 3. Results

### 3.1. Summative Overview of Participants

A total of 122 respondents participated in this study, with a proportionate distribution of 52% (n = 63) females and 48% (n = 59) males as shown in Table 1.

The sample in this consisted mostly of those aged 45–54 years (45%) (n = 55) and those aged over 55 years (45%) (n = 55), with very few who were aged below 44 years (10%) (n = 12). This is so because of the nature of the survey, which targeted people who were taking hypertension medication, and these were mostly older people. The data reported in the tables below, within which each row represents a specific category or factor, and each column represents the extent to which participants indicated their response. The percentages provided in the tables indicate the proportion of participants falling into each category and the extent of possible barriers to adherence to hypertension medication.

### 3.2. Possible Barriers to My Adherence to Hypertension Medication

Table 2 shows responses of the participants regarding their awareness as a possible barrier that undermined adherence to hypertension medication amongst study participants. In this, it was established that 34.4% (n = 42) of participants indicated that they were not aware why they needed to take hypertension medication, with 8.2% (n = 10) not aware to a large extent and 26.2% (n = 32) to a moderate extent. In addition, it was established that financial constraints were also a potential barrier to accessing medication, with 8.2% (n = 10) reporting a large extent, 27.9% (n = 34) a moderate extent, 49.2% (n = 60) a small extent, 1.6% a very large extent, and 13.1% (n = 16) a very small extent.

In addition, data presented in Table 2 revealed that forgetfulness was a possible barrier to adherence, as most of the participants, 42.6% (n = 52), indicated that they experience this challenge to a large and very large extent. This proportion is higher than the 27.8% (n = 34), this being a barrier to a small and very small extent. 29.5% (n = 36) revealed that they sometimes forget their medication. Cultural conflict was also an undermining factor with 18% (n = 22) primarily indicating it to a large extent, 19.7% (n = 24) a moderate extent, 42.6% (n = 52) a small extent, 1.6% (n = 2) a very large extent, and 18% (n = 22) a very small extent. Preference for traditional medicine was reported with 13.1% (n = 16) indicating a large extent, 27.9% (n = 34) a moderate extent, 32.8% (n = 40) a small extent, 8.2% (n = 10) a very large extent, and 18.0% (n = 22) a very small extent. Doubts about hypertension diagnosis resulted in 26.2% (n = 32) reporting a large extent, 27.9% (n = 34) a moderate extent, 29.5% (n = 36) a small extent, 1.6% (n = 2) a very large extent, and 14.8% (n = 18) a very small extent.

The dislike of side effects, however, sometimes, and on a few times to rarely, was a possible barrier to participants. In this, the majority, 73.8% (n = 90), of participants revealed that this was a possible barrier to a small and very small extent, and sometimes (moderate extent). Comparatively, only a smaller minority of 26.3% (n = 32) viewed this as a possible barrier to a large and very large extent. Language barriers with healthcare service providers accounted for the majority, 42.6% (n = 52) reporting it as a barrier to a smaller extent compared to 16.4% (n = 20) to a large extent. Interference of alcohol or drug use as a possible barrier resulted in 29.5% (n = 36) of participants reporting it to a moderate extent and 31.1% (n = 38) to a small extent. This compared favorably against 11.5% (n = 14) reported to a large extent and 0% to a very large extent. It was established that difficulty taking medication when away from home was a potential barrier to a small extent, 47.5% (n = 58), and 29.5% (n = 36), moderate extent, compared to 6.6% (n = 8) to a large extent. Another potential factor undermining adherence was feeling better and discontinuing medication. However, this was a potential barrier to a small extent, as revealed by the majority, 45.9% (n = 56) of respondents, when compared to 13.1% (n = 16) to a large extent. Waiting in long queues to receive medication was a possible barrier to a lesser extent to the majority of the participants, as a total of 45% (n = 55) viewed this to be a challenge to a small and very small extent, whilst 35% (n = 43) revealed that this happened sometimes. Overall, the findings showed that a significant portion of the participants had a lack of awareness regarding the reasons for taking their hypertension medication. Financial constraints also posed a potential barrier to medication access for most participants. Forgetfulness was a common issue, with a significant percentage of participants admitting to occasionally forgetting to take their medication. Cultural conflict and a preference for traditional medicine were also factors that affected adherence to hypertension medication. Additionally, doubt about their hypertension diagnosis was prevalent among a substantial number of participants.

### 3.3. Ranking the Possible Barriers to Medication for Hypertension

Table 3 outlines the ranking of possible barriers affecting adherence to hypertension medication. For example, on the complexity of the medication regimen, the majority, 39.3% (n = 48) of the population viewed it as the second most important barrier. The majority of participants also reported that the second most important barrier is having to take too many pills, 47.5% (n = 58), occasional failure to afford travel to get medication, 49.2% (n = 60), stigma and/or discrimination, 47.5% (n = 58). With regards to the affordability of travel to collect medication, it was established that the second highest number of study participants, 27.9% (n = 34), revealed this to be the third most important barrier, followed by 21.3% (n = 26) who said that it was the first important barrier. Nonetheless, the majority, 49.2% (n = 60), revealed that affordability was the second most important barrier.

The majority of respondents in this study, 45.7% (n = 56), reported stigma and/or discrimination for taking hypertension medication as the second most important barrier, after 27.9% (n = 34) who reported this to be the first most important barrier. The same pattern was also reported for the frequency of medication intake and fatigue in taking medication, with the majority 49.2% (n = 60) of study participants viewing this to be the second most important impediment, followed by 29.5% (n = 36) who saw it as the first most important factor, and 19.7% (24) who saw it as the third most important stumbling block. An almost similar pattern was reported for appointments with a healthcare provider interfering with other activities. On this, most study participants, 52.5% (64), revealed that this was the second most important barrier, followed by 24.6% (n = 30) who saw it as the first most important factor, and then 21.3% (n = 26) of those who viewed it as the third most important barrier.

On hypertension medication sometimes not being available, it is reported in Table 3 that the highest proportion of study respondents 60.7% (n = 74) revealed that this was the second most important barrier, compared to 21.3% (n = 26) who viewed it as the first most important factor, and 16.4% (n = 20) who saw it as being the third most important hindrance. A similar pattern was observed in the lack of family/social support, where most 41.0% (n = 50) participants revealed that this was the second most important hindrance, followed by 34.4% (n = 42) who viewed it as the first most important barrier. A similar pattern followed for lack of sufficient access to food, with the second most important barrier being viewed by many, 57.4% (n = 70), as the second most important barrier. However, the second highest proportions for doubts over diagnosis by the healthcare provider, 24.6% (n = 30), and advice not to take medication from people 26.2% (n = 32) constituted the second highest proportion of participants who viewed these to be the second most important barriers.

## 4. Discussion

### 4.1. Lack of Awareness and Financial Constraints

The findings from the Edenvale study, South Africa, suggest that most hypertensive adult patients in private healthcare have a lack of awareness about the need to take their hypertension medication. A cross-sectional survey carried out in South Africa suggests that the lack of awareness can be caused by a number of factors, which include insufficient knowledge about the condition itself, the importance of medication adherence, and potential side effects. Of concern, however, is that the reported awareness of the hypertensive status differed according to sex, with study findings showing that treatment and awareness were lower in men compared to women. This study also noted that discrepancies in treatment and awareness of hypertension between genders have previously been reported in research findings in the United States, where it was revealed that hypertensive men (20%) were generally much less aware of hypertension when compared to women (32%) with elevated blood pressure [23]. Non-adherence to anti-hypertensive medication due to a lack of awareness can only sustain the increasing prevalence and undermine the realization of desired health outcomes, particularly in men. In this regard, there is a need for education and awareness campaigns to promote health, targeting mostly men within communities and health establishments. To achieve this, China is implementing the “Healthy China 2030” planning outline, the first long-term strategic plan in the health sector at the national level in China. This plan establishes a “Big Health” and “Big Hygiene” concept focused on promoting health across the entire lifespan. Under this guidance, the “Healthy China Action (2019–2030)” includes cardiovascular disease prevention actions with goals set for 2022 and 2030. These goals include reducing cardiovascular mortality to 209.7 per 100,000 and 190.7 per 100,000 by 2022 and 2030, respectively. Additionally, the awareness rate of hypertension among residents aged 30 and above was set to be at least 55% by 2022 and 65% by 2030. In line with this strategy, the standardized management rate of hypertension patients should not be less than 60% and 70%, respectively, with continuous improvement in treatment and control rates. Additionally, societal and governmental requirements include the comprehensive implementation of blood pressure measurement at the first medical visit for individuals aged 35 and above. Primary healthcare institutions are expected to provide standardized health management services to residents aged 35 and older within their jurisdiction who have been diagnosed with essential hypertension. Moreover, there is an ongoing effort to promote the integrated management of the “three highs” (hypertension, hyperglycemia, and hyperlipidemia), including the assessment and intervention guidance for high-risk populations with overweight/obesity, elevated blood pressure, elevated blood sugar, and abnormal lipid profiles. This standardized approach may help address the lack of awareness so as to enable prevention and treatment for favorable health outcomes [24].

Financial constraints are also a significant potential barrier to access to medication for the majority of hypertensive patients [25]. Direct Out-Of-Pocket Payments (DOOPPs) are among the most important financing mechanisms in many health systems, especially in developing countries, adversely affecting equality and leading vulnerable groups to poverty [26]. Patients facing financial strain are more likely to experience medication non-adherence due to costs associated with medication, healthcare visits, and transportation. This can lead to poorer blood pressure control, increased risk of hypertensive crises, and higher healthcare costs in the long run [25]. Affordability is also reported as a barrier to hypertension and diabetes management in a study of Argentina. In addition, a study of Nepal also reports the lack of affordable services as a barrier undermining the treatment and control of hypertension in Nepal. This may mean that financial hindrance remains a significant barrier undermining the treatment of hypertension in healthcare settings. This, in turn, adds to the increasing burden of hypertension within health systems, which demands urgent attention from public health policy makers [27,28,29]. Several strategies have been employed, including reducing out-of-pocket costs, implementing public funding, improving financial management, and strengthening Primary Health Care (PHC). In this regard, the focus for public health policy makers needs to be on urgently working towards re-engineering Primary Healthcare towards well-functioning health systems care, particularly in Low-and Middle-Income settings [26,30,31]. To achieve this, the Lancet Global Health Commission on Financing Primary Health Care is of the view that all health systems need to commit to investing in Primary Health Care (PHC). To achieve this, this commission prescribes that healthcare systems need to come up with health funding plans and models that enable the mobilization, allocation, protection, and control of public health funds in a manner that is people and community-oriented, not hospital or specialist-based, and protects the population from financial health risk for equity in health care. However, it is noted that Primary Healthcare Re-engineering may achieve its goals through pragmatic, resourceful, and tactful health system governance, political commitment and support, as well as sector and stakeholder engagement [30]. This may help enhance access, affordability, and overall efficiency within the healthcare system, for favorable health outcomes, not only for hypertension [26,30,31].

### 4.2. Forgetfulness, Fear of Side-Effects, Perception, and Potential Solutions

Forgetfulness is another commonly reported barrier undermining adherence to hypertension medication. The study of Edenvale, South Africa, reports a significant percentage of participants, 43.7% (n = 53), admitting to occasionally forgetting to take their medication as prescribed. Forgetfulness was reported in a qualitative study of barriers and facilitators of habit building for long-term adherence to anti-hypertensive therapy among people with hypertensive disorders in Los Angeles, California. This Los Angeles study reports that forgetfulness is a barrier to habit formation towards medication, which emerges due to unstable anchoring, routine disruptions, novelty, and perceived need. To address this challenge, participants in this study proposed enablers of habit formation. These enablers included predictable standard daily routines within which to anchor the uptake of medication, which include the time of the day and the location of taking medication on each day. In this, a number of participants in this study (n = 8; 40%) revealed in detail a series of daily activities to show how they had incorporated the uptake of medication into their day-to-day routine. Most of the participants (n = 10; 50%) resolved to make use of stable anchors and reminders unlikely to be disturbed by other factors. In this regard, it was reported that the most common anchors used included the times of waking up in the morning, the first meal early in the day, lunch and supper, and the already existing times at which to take other medication. A few (n = 3; 15%), however, revealed some reminders that enabled their caregivers and/or pets to help remind them when the time to take their medication arrived. This study also reports that home was the most common place where medication is taken, particularly in the bathroom or kitchen, where the patients carry out routine activities. As such, having one to keep their medication in these places will always help provide a visual reminder each time. In addition, other places to keep medication included places of work and inside the car, particularly if the patient is prescribed to take part of their medication in the afternoon or when away from home. Most of the healthcare providers (n = 5.71%) in this study were of the view that anchoring the uptake of medicine to pre-existing daily rituals was the strongest intervention to enable habit formation. This, in their view, can complement other ongoing interventions, which include counselling sessions with each patient (n = 6.86%) and the application of visual reminders (n = 3.43%) as strategies to help them develop a habit of taking their prescribed medication [32].

Perhaps forgetfulness within patients may also emerge out of doubt or skepticism about their hypertension diagnosis. The Edenvale study in South Africa reveals that doubt or skepticism is prevalent among a substantial number of patients, with 71.2% (n = 87) reporting that this doubt led to non-adherence. Studies report that many hypertensive patients do not adhere to anti-hypertensive medication because they have a wrong perception towards hypertension, or they are not confident in their anti-hypertensive medication and fear potential side effects. To address this, a qualitative study conducted in the catchment area of two semi-urban clinics in Dar es Salaam, Tanzania, to determine the perceived barriers to hypertension medication adherence proposes phone text messaging as a potential reminder intervention system by both patients and healthcare providers to mitigate forgetfulness. Standard mobile telephone text messages, including WhatsApp, with support from medical teams such as the Community Centre for Preventive Medicine (CCPM) center and other patient-focused follow-up routines within the community, market areas, and churches in collaboration with local healthcare officials, may be used to help remind patients about medication uptake in order to help improve adherence. The Tanzania study further notes that these interventions need to be reinforced through results-based monitoring and evaluation for the effective control of hypertension at the patient and healthcare provider levels. Patient education strategies also help patients demystify any doubts about their diagnosis and address negative perceptions towards antihypertension medication [33].

### 4.3. Discontinuation and Cultural Conflict

Feeling better and therefore discontinuing medication may also contribute to non-adherence and/or discontinuation of medication [34,35,36]. Feeling better can indeed contribute to medication non-adherence and discontinuation. When individuals experience symptom relief, they may mistakenly believe they are cured and no longer need the medication, leading to premature cessation of treatment. Discontinuation may also be caused by misinterpretation of improvement, lack of awareness of the long-term nature of treatment, negative attitudes towards medication, and underestimation of relapse risk [37,38].

Cultural conflict and a preference for traditional medicine are also factors that impact the participants’ adherence to their hypertension medication. The study of hypertensive patients in Edenvale, South Africa, reveals that taking medication is seen as causing a cultural conflict for 57.3% (n = 70) of participants, and a preference for traditional medicine is expressed by 59.2% (n = 72) of participants. However, a systematic review of literature into the health beliefs and medication adherence in patients with hypertension notes that patients’ beliefs and their relationship to medication adherence appear to vary unpredictably across and within countries. This may mean that there remains a need for specific studies exploring the correlation between patient beliefs and their relationship to medication adherence within and across countries [39].

### 4.4. The Challenge of Language, Alcohol, and/or Drug Use

Language with healthcare service providers is also reported to be a significant barrier, with more than half of the participants, 56% (n = 68), in the study of patients receiving private healthcare in Edenvale, South Africa, having trouble understanding the language used, and 21% (n = 26) experiencing great difficulty in understanding the language used. This view is supported by a study into the disparities in hypertension associated with Limited English Proficiency (LEP), which reports that LEP is associated with poor health status and worse outcomes, with those with LEP having higher odds of elevated blood pressure on physical examination [40]. Language as a barrier may be further compounded by the complexity of the medication regimen. This includes finding the medication regimen too complicated, or having to take too many pills every day, as the study of Edenvale, South Africa, shows.

Alcohol and/or drug use is reported to interfere with medication adherence in the study of patients from Edenvale, South Africa, even though this was to a small extent. However, a pooled analysis of 22 million patients in the United States reports that heavy alcohol consumption was associated with a decline in adherence, which may contribute to an increasing burden of hypertension [41].

### 4.5. Stigma and Discrimination, and Future Research

Stigma and discrimination related to taking the medication are reported by most of the participants in the study of Edenvale, South Africa, as a factor that further impacts adherence. Stigma is a well-documented barrier to health-seeking behavior, engagement in care, and adherence to treatment across a range of health conditions globally. A study of hypertension Stigma among Black Women reports that stigma can potentially deter adherence to high blood pressure treatment. The fear of being labeled or judged, especially due to perceived lifestyle factors associated with high blood pressure, can lead individuals to delay or avoid treatment, contributing to uncontrolled hypertension and its associated health risks [42]. To address this, a study carried out in Uganda suggests that family support is important in the management of hypertension. This support and assistance from family members may compel them to adhere to antihypertensive medication. This family support can be through helping remind the patient to take the dose of hypertension medication when due throughout the day, counseling about the need to take medication, facilitating access to healthcare by providing transport, and the money required for healthcare services and medicine [43]. Future research may focus on why certain barriers are prevalent in the specific context of private health facilities in South Africa and how this may differ between the public and private health systems.

### 4.6. Limitations

Edenvale is generally an upper-middle-income area in which people can afford private healthcare, and as such, we assumed that all participants had access to the internet and with potential heterogeneous digital literacy. However, we acknowledge that this may also have been a limitation for other potential participants without internet access and/or digital literacy living in this area who may have wanted to take part in this study. Further statistical analysis on this subject is planned for future research and publications. Future research in Edenvale and in other middle-income areas will include hypertensive patients in both public and private healthcare.

## 5. Conclusions

This cross-sectional study of Edenvale provides evidence showing that a significant portion of the hypertensive patients in private healthcare have a lack of awareness regarding the reasons for taking their hypertension medication, which may be associated with lower adherence. Financial constraints are also associated with limited access to medication, which in turn may undermine adherence amongst most patients. Forgetfulness is a common challenge associated with a significant percentage of patients admitting to occasionally forgetting to take their medication. Additionally, doubt about hypertension diagnosis is linked to a high prevalence of non-adherence to anti-hypertensive medication in a substantial number of patients. This may be compounded by the views that the medication regimen is too complicated, or that they feel overburdened by having to take too many pills every day, in addition to concerns about complex medication regimens resulting in fatigue from medication intake, all of which are linked with non-adherence.

These challenges were further compounded by problems in accessing healthcare facilities for medication, difficulty in following up with healthcare practitioners, perceived incorrect diagnosis, and cultural issues related to lack of social support from family and friends, factors which are associated with non-adherence to medication. Cultural conflict and a preference for traditional medicine were also identified as factors associated with low adherence to hypertension medication. This study underscores the need to understand the barriers that undermine adherence to anti-hypertensive medication, so as to help respond accordingly for positive health outcomes in order to reduce the burden of hypertension on healthcare systems, towards achieving Sustainable Development Goal (SDG) 3 on health and care for all.

## Figures and Tables

**Table 1:** Demographic Profile of Participants.

Sex	Percentage (*%*)	Numbers (n)
Males	0.48	59
Females	0.52	63
Total	100	122

**Table 2:** Possible barriers undermining adherence to hypertensive medication.

	Very Large Extent (Most of the Time)	Large Extent (All the Time)	Moderate Extent (Sometimes)	Small Extent (a Few Times)	Very Small Extend (Rarely)
	(%) (n = 122)	(%) (n = 122)	(%) (n = 122)	(%) (n = 122)	(%) (n = 122)
Lack of awareness	3.3 (n = 4)	8.2 (n = 10)	26.2 (n = 32)	27.9 (n = 34)	34.4 (n = 42)
Financial constraints	1.6 (n = 2)	8.2 (n = 10)	27.9 (n = 34)	49.2 (n = 60)	13.1 (n = 16)
Forgetfulness	1.6 (n = 2)	16.4 (n = 20)	29.5 (n = 36)	26.2 (n = 32)	26.2 (n = 32)
Conflict with culture	1.6 (n = 2)	18.0 (n = 22)	19.7 (n = 24)	42.6 (n = 52)	18.0 (n = 22)
Preference for traditional medicine	8.2 (n = 10)	13.1 (n = 16)	27.9 (n = 34)	32.8 (n = 40)	18.0 (n = 22)
Doubts about hypertensive condition	1.6 (n = 2)	26.2 (n = 32)	27.9 (n = 34)	29.5 (n = 36)	14.8 (n = 18)
Side-effects of hypertensive medication	6.6 (n = 8)	19.7 (n = 24)	36.1 (n = 44)	27.9 (n = 34)	9.8 (n = 12)
Language as a barrier	4.9 (n = 6)	16.4 (n = 20)	23.0 (n = 28)	42.6 (n = 52)	13.1 (n = 16)
Alcohol and/or drug use	0 (n = 0)	11.5 (n = 14)	29.5 (n = 36)	31.1 (n = 38)	27.9 (n = 34)
Difficulties of medication uptake when away from home	1.6 (n = 2)	6.6 (n = 8)	29.5 (n = 36)	47.5 (n = 58)	14.8 (n = 18)
Discontinuation	0 (n = 0)	13.1 (n = 16)	27.9 (n = 34)	45.9 (n = 56)	13.1(n = 16)
Long queues	5.0 (n = 6)	15.0 (n = 18)	35.0 (n = 43)	31.7 (n = 39)	13.3 (n = 16)

**Table 3:** Ranking of possible barriers to adherence to hypertensive medication.

	First Most Important Barrier	Second Most Important Barrier	Third Most Important Barrier	Missing
	(%) (n = 122)	(%) (n = 122)	(%) (n = 122)	(%) (n = 122)
Complications of the medication regimen	34.4 (n = 42)	39.3 (n = 48)	24.6 (n = 30)	1.6 (n = 2)
Too many pills every day	26.2 (n = 32)	47.5 (n = 58)	24.6 (n = 30)	1.6 (n = 2)
Lack of affordability	21.3 (n = 26)	49.2 (n = 60)	27.9 (n = 34)	1.6 (n = 2)
Stigma and/or discrimination	27.9 (n = 34)	47.5 (n = 58)	23.0 (n = 28)	1.6 (n = 2)
Taking medication too often	29.5 (n = 36)	49.2 (n = 60)	19.7 (n = 24)	1.6 (n = 2)
Fatigue	29.5 (n = 36)	49.2 (n = 60)	19.7 (n = 24)	1.6 (n = 2)
Appointments with healthcare service provider interfering with other activities	24.6 (n = 30)	52.5 (n = 64)	21.3 (n = 26)	1.6 (n = 2)
Limited medication availability	21.3 (n = 26)	60.7 (n = 74)	16.4 (n = 20)	1.6 (n = 2)
Lack of family/social support	34.4 (n = 42)	41.0 (n = 50)	23.0 (n = 28)	1.6 (n = 2)
Insufficient access to food	23.0 (n = 28)	57.4 (n = 70)	18.0 (n = 22)	1.6 (n = 2)
Doubt	21.3 (n = 26)	52.5 (n = 64)	24.6 (n = 30)	1.6 (n = 2)
Social influence	23.0 (n = 28)	45.9 (n = 56)	26.2 (n = 32)	4.9 (n = 6)

## Data Availability

The data that support the findings of this study are available in a de-identified format from the corresponding author upon reasonable request, particularly the need to protect the privacy and confidentiality of study participants as agreed when informed consent was granted.

## Abbreviations

AU	African Union
BP	Blood Pressure
CVD	Cardiovascular Disease
CD	Cardiovascular Disorder
CCPM	Community Centre for Preventive Medicine
DALYs	Disability Adjusted Life Years
DOOPPs	Direct Out-Of-Pocket Payments
HICs	High Income Countries
LEP	Limited English Proficiency
LMICs	Low-and Middle Income Countries
NCDs	Non Communicable Diseases
NHREC	National Health Research Ethics Committee
PASCAR	Pan African Society of Cardiology
PHC	Primary Health Care
RACG	Risk Assessment Collaborating Group
REC	Research Ethics Committee
SA	South Africa
SDG	Sustainable Development Goal
SMS	Short Message Service
SPSS	Statistical Package for Social Sciences
SSA	Sub Saharan Africa
UN	United Nations
WHO	World Health Organization
